# Effects of biopolyimide molecular design on their silica hybrids thermo-mechanical, optical and electrical properties†

**DOI:** 10.1039/c8ra01965g

**Published:** 2018-04-16

**Authors:** S. Dwivedi, S. Sakamoto, S. Kato, T. Mitsumata, T. Kaneko

**Affiliations:** Graduate School of Advanced Science and Technology, Energy and Environment Area, Japan Advanced Institute of Science and Technology 1-1 Asahidai Nomi Ishikawa 923-1292 Japan kaneko@jaist.ac.jp; Japan Science and Technology, ALCA Tokyo 102-0076 Japan; Department of Materials Science & Technology, Faculty of Engineering, Niigata University Ikarashi, Nishi-ku Niigata 950-2181 Japan

## Abstract

Polymers, derived from bio-derived resources, have gained considerable momentum because of a lower dependence over conventional fossil-based resources without compromising the materials' thermo-mechanical properties. Unique characteristics of organic and inorganic materials can be incorporated by a combination of both to obtain hybrid materials. We have recently developed a series of transparent biopolyimides (BPI) from a biologically derived exotic amino acid, 4-aminocinnamic acid (4ACA) to yield 4-amino truxillic ester (4ATA ester) derivatives. In the present research, the polyimide-precursor was subjected to sol–gel polycondensation reactions with silicon-alkoxide followed by annealing under *vacuo* to yield a biopolyimide-silica hybrid. The biopolyimide structures (4ATA acid/ester) and their silica hybrids thermo-mechanical, electrical and optical performance were evaluated. It was found that the biopolyimide with 4ATA(ester) yields thermo-mechanically robust films with very high electrical stability as well as optical transparency, plausibly due to the uniform dispersion of the silica particles in the biopolyimide matrix.

## Abstract 2

Biopolyamide structure and their silica hybrids performances were studied. Biopolyamide with inability to interact with silanol during sol–gel condensation for silica formation showed superior thermo-mechanical, optical and electrical properties.

## Graphical abstract

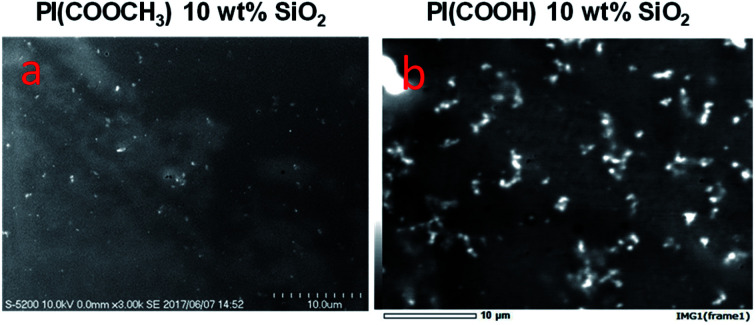

## Introduction

“Functional polymers” and their “synergistic interaction” with a hybrid precursor are the key issues to realize high performance polymer hybrids.^1–3^ The term here, functional polymers, represents the type(s) of active functional groups, while the synergistic interaction dictates the molecular interaction between the hybrid molecules and the polymer units during hybridization. Most of the polymer hybrids have been invented and developed mainly in an empirical manner from the viewpoint of performance optimization in terms of thermo-mechanical, optical, electrical performances, and so on.^4–9^ In most cases, the roles of active functional groups of the polymer and their impacts on the various kinds of performances are only roughly understood. The difficulty comes from several reasons: a polymer performance is usually affected by several chemical and structural factors in a complicated way, while to vary one of these factors without changing the other factors demands systematic synthetic techniques.^10–14^

High-performance polymers are used in applications demanding service at enhanced temperatures, while maintaining their structural integrity and an excellent combination of chemical, physical, and mechanical properties.^15^ Among them, aromatic polyimides represent an increasing important class of materials in aerospace, microelectronics, and other industrial applications.^16–19^ Polyimides are one of the important classes of polymers used as interlayer dielectrics for advanced printed circuit boards and multichip module packing. Dielectric materials used for thin film multichip modules must meet a number of material and electrical requirements including a low-dielectric constant, low-dissipation factor, minimal moisture absorption, and high thermal stability.^20–23^ Many desirable properties of polyimides, including good thermo-oxidative stability and excellent mechanical properties, contribute to their success. However, the polyimides exhibit relatively high dielectric constant. A way to obtain materials with improved properties is to form organic–inorganic hybrids by incorporating inorganic fillers. The hybrid polyimides represent a class of new generation of materials that combine the properties of the ceramic phase with those of organic polymers.^24^ The incorporation of various metallic additives, such as TiO_2_, BaTiO_3_, Al_2_O_3_, SiO_2_ and, ZnO into polyimides has been reported to improve the properties of the resulted materials.^25–27^ However, the importance of polyimide microstructure and the performance of their hybrids have been rarely systematically studied.

The sol–gel technique is an excellent method to produce hybrid polyimide/silica polymers. This method consists hydrolysis of an alkoxysilane and a polycondensation process.^28,29^ Therefore, the processing of the polyimides is generally carried out *via* soluble poly(amic acid) precursors, which are cast onto various substrates, and then they are converted into polyimide films by thermal treatment. The good solubility of the poly(amic acid) precursors in amidic solvents makes possible the introduction of metal oxide precursors into their solutions. Also, the excellent thermal stability of polyimides makes possible the required thermal treatment up to high temperatures without inducing appreciable degradation in the organic phase. Lee *et al.*, found that the phase separation occurred at higher silica content leads to the lower mechanical strength of the resulting film relative to the pure polyimide or lower silica content containing polyimide.^30,31^ Therefore, the compatibility between the polyimide and silica should be improved by precise monomer molecular design.

Recently, we have established the syntheses of a diamine from biologically obtained exotic amino acid, 4-aminocinnamic acid (4ACA) to yield 4,4′-diamino truxillic ester (4ATA ester), used for the biopolyimide preparation.^32,33^ In this article, we report the preparation of a new diamine 4,4′-diamino truxillic acid and a series of biopolyimide and their silica hybrids. The affinity of silica phase with the polymeric matrix, was controlled with the acid/ester functionalization of the diamine moiety in the biopolyimide. The biopolyimide structural characteristics and the thermo-mechanical, electrical and the optical performances were correlated.

## Experimental

### Materials

Dianhydride, 1,2,3,4-tetracarboxycyclobutane dianhydride (CBDA, Tokyo Chemical Industries (TCI), Tokyo, Japan) was purified by sublimation under reduced pressure before use. Diamine precursor 4-aminocinnamic acid (4ACA, TCI, Tokyo, Japan), esterification reagent trimethylsilyl chloride (TMSCl, Sigma-Aldrich, Tokyo, Japan), and polymerization solvent super-dehydrated *N*,*N*-dimethylacetamide (DMAc, Kanto Chemical Corporation, Tokyo, Japan) and solvents were used as received. All chemicals used were of research grade.

### Syntheses

Monomers were synthesized with the Scheme S1.† 4,4′-Diamino-α-truxillic acid (4ATA) dihydrochloride was synthesized by the drop-wise addition of 12 N hydrochloric acid solution (5.6 ml) in the solution of 4ACA (2.0 g, 12.4 mmol) in acetone (30 ml) to produce 4-aminocinnamic acid hydrochloride (1.74 g, 4.35 mmol). The obtained product was subjected to irradiation by a 100 W high pressure Hg-lamp (Omni Cure S1000, EXFO Photonic Solution Inc.) with a 250–450 nm band-pass filter with an intensity of 2.7 mW cm^−3^ for 24–36 h to induce [2 + 2] photocycloaddition. The reaction was monitored by ^1^H NMR (400 MHz, DMSO-d_6_, *δ*, ppm) by disappearance of peak for the olefinic protons: 3.82 (dd, 2H, *J* = 7.7, 9.6 Hz), 4.30 (dd, 2H, *J* = 7.7, 9.6 Hz), 7.33 (d, 4H, *J* = 7.7 Hz), 7.45 (d, 4H, *J* = 7.7 Hz), 10.37 (s, 6H), 12.07 (s, 2H).

The obtained 4,4′-diamino-α-truxillic acid (4ATA) dihydrochloride was subjected to the esterification. A solution of 4 ATA (1.71 g, 4.27 mmol) in methanol was prepared and TMSCl was added drop-wise. The reaction was monitored by ^1^H NMR (400 MHz, DMSO-d_6_, *δ*, ppm) and allowed to takes place 12 h. The obtained product was filtered and dried at 40 °C under *vacuo* for 6 h. The dimethyl ester of 4ATA salt (1.83 g, 4.28 mmol) was dissolved in water and neutralized by 1 N NaOH solution to obtain 4,4′-diamino-α-truxillic dimethyl ester (1.29 g, 3.64 mmol). The obtained product was dried and subjected to soxhlet purification using ethyl acetate to get crystals. The purified 4,4′-diamino-α-truxillic dimethyl ester was confirmed using ^1^H NMR (400 MHz, DMSO-d_6_, *δ*, ppm).

**Scheme 1:**
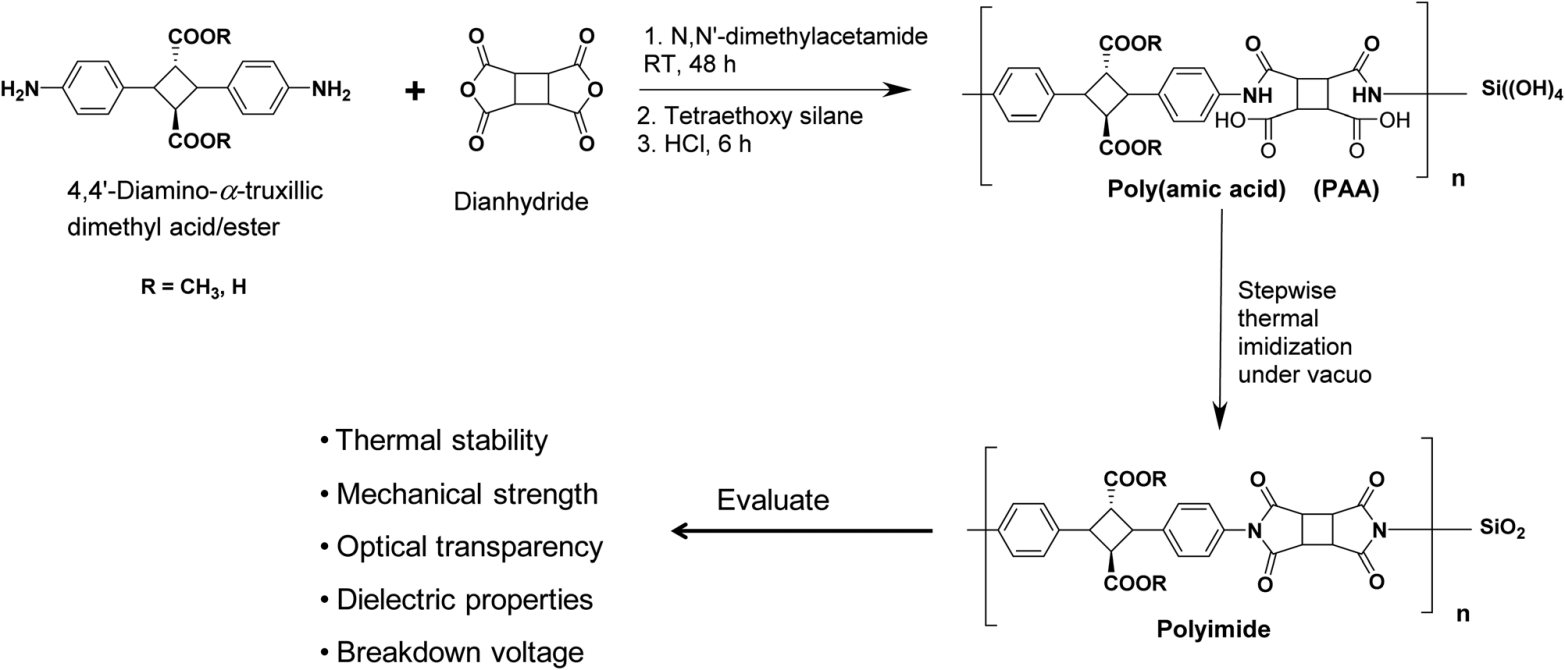
Polymerization reaction scheme and the resulting biopolyimide/silica hybrids evaluated properties.

The crystals of 4,4′-diamino-α-truxillic dimethyl ester, (0.20 g, 0.5647 mmol) was dissolved in DMAc (0.8 ml, 9.6 mmol) under a nitrogen atmosphere followed by the addition of CBDA (0.11 g, 0.5647 mmol), as shown in the Scheme 1. The reaction mixture was stirred at room temperature to produce a clear to pale yellow viscous poly(amic acid) (PAA) solution in 48 h. The PAA solution was diluted in DMAc, and then added dropwise in methanol/water mixture to precipitate PAA fibers, which were filtered and dried under *vacuo* at 60 °C for 12 h. The PAA film was obtained by casting a PAA homogeneous solution in DMAc onto a glass plate and heating at 75 °C. The obtained PAA films were subjected to a thermal imidization in an oven under reduced pressure by stepwise heating at 100, 150 and 200 °C for 1 h, 1 h and 3 h at each step respectively to obtain the polyimide films. Similar procedure for the polyimide preparation has been followed with other dianhydride structures.

Biopolyimide-silica hybrids were synthesized by the sol–gel condensation. Pre-calculated amount of TEOS was added in the poly(amic acid) solution in the DMAc followed by the addition of HCl (catalyst) to yield the silica formation the polymer matrix. Gradual annealing of the resulting poly(amic acid)/silica films up to 200 °C results the formation of the biopolyimide-silica hybrids.

### Characterization

The number-average molecular weight (*M*_n_), weight-average molecular weight (*M*_w_) and the molecular weight distribution (PDI) were determined by gel permeation chromatography (GPC, concentration 5 g L^−1^, DMF eluent, Shodex SB-800HQ, Showa Denko K.K., Tokyo, Japan) after calibration with pullulan standards.

NMR measurements were performed by a Bruker Biospin AG 400 MHz, 54 mm spectrometer using DMSO-d_6_ as the solvent. The FT-IR spectra were recorded with a Perkin-Elmer Spectrum One spectrometer between 4000 and 600 cm^−1^.

Thermogravimetric analysis (TGA) was performed on STA 7200 (Hitachi, Tokyo, Japan) at a heating rate of 10 °C min^−1^ under a nitrogen atmosphere. The polymer specimens were dried at 100 °C for 1 h to remove any absorbed moisture before both TGA. The tensile measurements were carried out at an elongation speed of 0.5 mm min^−1^ on a tensiometer, the Instron 3365 at room temperature.

The ultraviolet-visible (UV-vis) optical absorption spectra were recorded on a Perkin-Elmer, Lambda 25 UV/vis spectrophotometer at room temperature over the range of 200–800 nm. X-ray diffraction (XRD, Rigaku SmartLab, Tokyo, Japan) pattern was used to determine the crystallinity degree of the polyimide films. The morphology and the elemental composition of the polyimide films were determined by scanning electron microscope (SEM, JEOL Japan).

The dielectric constant for the biopolyimide (BPI) and their silica hybrids was measured by using a LCR meter (IM 3536 HIOKI, Nagano, Japan) at 293 K. An electric voltage of 1.0 V was applied by two electrodes made up of brass. The frequency was kept at 1 MHz.

The dielectric breakdown voltage was measured by using an electrical safety analyzer (SE7430 Keisoku Giken, Yokohama, Japan) at room temperature. The electric voltage was applied by two terminals method up to 6 kV for DC with a ramp-up time of 120 s and with a rate of 1 kV s^−1^. The electrode was made of stainless steel with a diameter of 20 mm. In the process, the samples were cut to dimensions of 40 × 40 mm. The electric breakdown (EBD) strength of the films was calculated by dividing the electric breakdown voltage by the thickness of samples.

## Results and discussion

### Material syntheses

Production of diamine from 4ACA demands the amalgamation of two precursor units. A [2 + 2] photocycloaddition reaction of *trans*-cinnamic acid, with radiations (*λ* > 260 nm) to yield α-truxillic acid. Olefinic functionality of 4ACA was utilized to obtain the corresponding α-truxillic acid from the 4ACA salt, characterized by ^1^H NMR (Fig. S1†). The reaction was monitored by the ^1^H NMR with the disappearance of the olefinic protons (7.44–7.40 and 6.15–6. 12) and development of cyclobutane proton signals (4.36–3.80) (Fig. S2†). Thereafter, α-truxillic acid salt was neutralized by 1 M NaOH and purified by activated charcoal to obtain 4ATA (acid type, Fig. S3†). On the other hand, as precise monomer design demands the protection of the two-carboxylic acid groups of 4ATA to avoid any possible interference during the polymerization. Therefore, esterification of 4ATA was performed with methanol and TMSCl, confirmed by the ^1^H NMR (Fig. S4†). Finally, the 4ATA-methyl ester salt was neutralized by 1 M NaOH aqueous solution and the final product was confirmed by ^1^H NMR (Fig. S5†).

The polycondensation was carried out in a 1 : 1 solution of 4ATA ester/acid and dianhydride in the super dehydrated DMAc.^21^ The development of viscosity with the progression of the reaction indicates the formation of poly(amic acid) (PAA). FT-IR analysis of the PAA fibrils shows a broad signal around 2600–3600 cm^−1^ (O–H, stretching), two sharp absorption bands at 1720 cm^−1^ (C–O stretching, carboxylic and ester) and 1670 cm^−1^ (C–O stretching, amide) and aromatic peaks at 1525 cm^−1^ and 1432 cm^−1^ (C–H first overtone, aromatic) (Fig. S6†). TEOS with catalyst (HCl) lead to the sol–gel condensation lead to the silica precursor formation. The number average molecular weight (*M*_n_) and PDI of the PAA was measured by GPC, which were found to be 4.7–5.2 × 10^5^ and 1.4 respectively. PAA films were prepared by solution casting and stepwise annealing yields polyimide films. The chemical structure of the PI was confirmed by the FT-IR. A signal at 1375 cm^−1^ and 1175 cm^−1^ (C–N stretching, imide) confirms the formation of imide ring and the imidization under annealing process. The peak at 1211 cm^−1^ has been assigned mainly to the C–O (stretch, COOH, COOCH_3_). Furthermore, the spectra shows two peaks for the carbonyl at 1785 cm^−1^ (C=O, asymmetric stretching) and 1716 cm^−1^ (C=O symmetric stretching). The obtained polyimide was confirmed to have high chemical resistance except for concentrated sulfuric acid.

### Optical properties

The neat biopolyimide (BPI) and their silica hybrid films were prepared and subjected to the UV-vis spectroscopy for understanding their optical properties. It was interesting to observe that the COOCH_3_ type BPI were more transparent than COOH type BPI. However, this gap in the transparency gets wider with the incorporation of silica in the BPI. The COOCH_3_ type BPI silica hybrids transparency improved with the silica incorporation. On the contrary, in the case of the COOH type BPI the transparency was reduced with silica incorporation, which may be attributes to the larger silica formation in the polymer matrix, responsible for the light reflection/scattering rather than transmission. In order to confirm the silica particle distribution homogeneity in the polymer matrix, SEM analyses was conducted over the film surface. It was observed that in the case of COOH type BPI, silica particles were present in the form of micro-meter sized particles. On the contrary, COOCH_3_ type BPI silica hybrids show rather more uniform surface. Thereby, we confirmed the silica particle formation in the COOH type BPI was more non-uniform than COOCH_3_ type BPI (except at over loading *ca*. 20 wt%). The silica particle dispersion was confirmed using the SEM analyses.

### Thermo-mechanical properties

Thermal stability of the BPI and their silica hybrids were studied by thermogravimetry, by heating sample from 25 °C to 800 °C at the rate of 5 °C min^−1^ under nitrogen. The degradation temperature of a material may be represented at various extent of material degradation. Mu *et al.*, has shown the importance of *T*_d1_ (1 wt% loss temperature) over other degradation temperatures.^34^ Considering the complexities of the thermal degradation, *T*_d1_ and *T*_d10_ (conventional degradation parameter in polymers) both have been mentioned in the Table 1. It was interesting to observe that both the *T*_d1_ and *T*_d10_ follows almost same trend for the BPI silica hybrids. It was observed that the COOH type BPI was more thermally stable than the COOCH_3_ type BPI at 5 wt% of silica loading. The greater stability of the COOH type BPI may be envisaged to the possibility of H-bonding interactions in the polymer matrix between COOH and other electron rich moieties (O, N) apart from the conventional charge-transfer (CT) interactions. Incorporation of silica in the BPI matrix leads to the decrease in the thermal stability for the COOH type. While, the COOCH_3_ type BPI silica hybrids thermal stability improved with the addition of silica, until 10 wt% and lead to a decrement in stability at 20 wt%. The observation may be attributed to the enhanced uniform synergistic chain-transfer interactions between the silica units and the COOCH_3_ BPI chains. The non-uniformity (or over-loaded) in the silica distribution leads to the weaker synergism for CT interactions between silica and COOH type BPI.

**Table 1:** Thermo-mechanical and optical properties of biopolyimide and their silica hybrids

Polyimide type	SiO_2_ content (wt%)	*δ* a (MPa)	*ε* a (%)	*E* a (GPa)	*T*_d1_ b (°C)	*T*_d10_ b (°C)	*T*_450 nm_ c (%)
COOH	5	110	3.9	4.1	326	403	85.7
	10	122	5.5	6.6	332	397	77.1
	15	111	5.2	5.8	296	354	75.4
	20	119	6.2	4.6	204	249	73.9
COOCH_3_	5	84	3.0	2.7	319	377	86.6
	10	112	7.0	3.5	323	381	88.3
	15	124	5.9	4.9	337	388	89.2
	20	149	5.6	5.6	315	376	90.2

a *δ*, *ε* and *E* refers to tensile strength, elongation degree and modulus of elasticity respective determined by the tensile tests.

b *T*_d1_ and *T*_d10_ represents the 1 wt% and 10 wt% thermal degradation temperature, respectively, measured under nitrogen through TGA.

c *T*_450 nm_ shows the transmittance at *λ* = 450 nm was measured by UV-vis spectroscopy.

**Fig. 1:**
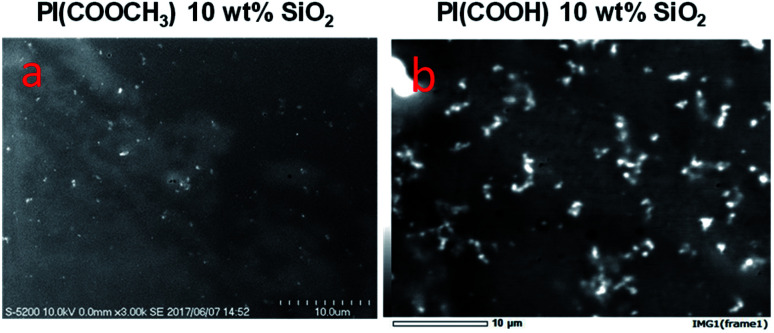
Biopolyimide/silica SEM micrographs at 10 wt% loading.

The BPI silica hybrids robustness was judged through the mechanical testing (Table 1). It was observed that the COOCH_3_ type BPI silica hybrids shows an increase in modulus of elasticity, degree of elongation and the tensile strength with the silica content in the polymer matrix. It was interesting to note that the tensile strength of the COOCH_3_ type BPI silica hybrid was increased by over 77% than the neat BPI. While the modulus of elasticity was increased over 107% and degree of elongation by 86% for 20 wt% of silica incorporation in the COOCH_3_ type BPI structure. On the contrary, in the case of COOH type BPI, it was observed that the mechanical properties increased but with a relatively random trend. It was observed that the tensile strength of the BPI increased by around only 9%. Moreover, modulus of elasticity and elongation degree improved by hardly 11% and 61% respectively. The higher tensile strength of the neat COOH BPI may be attributed to the more ordered structure of the BPI due to H-bonding in the polymer matrix. However, the slower increase in the COOH type PI silica hybrids mechanical properties can be a trait arose due to the disturbed H-bonding and the non-homogeneous silica particle distribution, which causes over stress along the particle grain boundary lead to easier film rupture. On the contrary, in the case of COOCH_3_ type BPI, the silica particles were more uniformly distributed (confirmed by SEM image, Fig. 1) along the polymer matrix and the stress was evenly shared along the matrix, which led to higher mechanical robustness development.

Furthermore, the biopolyimide crystallinity was investigated by X-ray diffraction (XRD). The XRD pattern of biopolyimide yields two diffraction peaks at 2*θ* = 6° and 17° overlapping with a broad amorphous halo (Fig. S7†). The XRD diagram of neat biopolyimide indicates a partial crystallization (14–22%). It is noteworthy to mention that the biopolyimide silica hybrids crystallinity decreased with the increase in the silica content, which may be envisaged as a reason for the improved elasticity of the biopolyimide-silica composites.

### Dielectric constant

Materials with low dielectric constants (low-*ε*) are required by the modern integrated circuits (IC) in order to reduce the resistance capacitance (RC) delay and minimize cross-talk noise. In this respect, polyimide (PI) is desirable because of its high thermal stability, excellent mechanical properties, and low dielectric constant.^35–38^ Therefore, recent researches are focused on reducing the dielectric constant of PI by synthesis of novel structures and organic–inorganic composites, especially using sol–gel methodology.^36^ Introduction of fillers into the PI matrix also can reduce *ε* considerably. For example, Zhang *et al.* and Kim *et al.* incorporated silica tubes into PI by self-assembly method to obtain a composite with a low value of *ε* (dielectric constant) up to 3.6–4.0.^39,40^ However, in spite of several attempts, the dielectric constant of PI composites is usually more than 3.5 which does not meet the stringent demand by the modern microelectronics industry.^41–44^

**Fig. 2:**
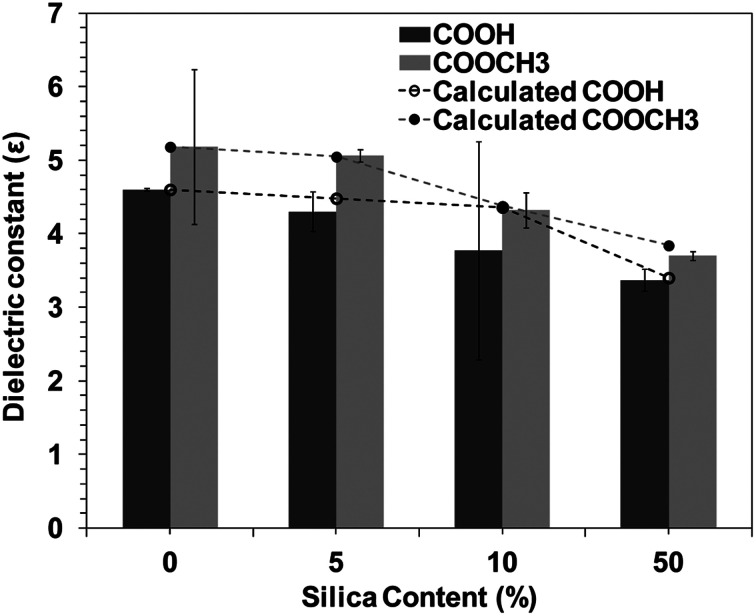
Dielectric constant variations with silica content in biopolyimide.

Fig. 2 shows the silica concentration dependence of dielectric constant for BPI hybrids. Dielectric constants of the BPI/silica hybrids decreased from 4.6 (neat COOH type BPI) to 3.3 (50 wt% silica). Neat COOCH_3_ type BPI possesses greater dielectric constant than COOH type BPI, which may be attributed to the inherent polarization of COOCH_3_ moiety. However, irrespective of kind of BPI, the dielectric constant almost linearly decreased with the increase in the silica loading, suggesting that the decrease in the dielectric constant is due to the low dielectric constant of silica. The dielectric constant can be explained by the following equation, which is a linear combination of dielectric constant of BPI and silica,

1 *ε*_hybrid_ = *ε*_Si_*φ*_Si_ + (1 − *φ*_Si_)*ε*_BPI_

where, *ε*_hybrid_, *ε*_Si_ and *ε*_BPI_ represents the dielectric constant for BPI/silica hybrid, silica, and biopolyimide respectively; *φ*_Si_ is the amount of silica. The line along the data in Fig. 2 represents the result of fitting by eqn (1). The dielectric constant for both biopolyimides was well fitted by eqn (1), suggesting that the interfacial polarization effect is less in the hybrids. The values of *ε*_Si_ were determined to be 2.6 for COOH and 3.5 for COOCH_3_, which were close to the literature values of silica.^45^

### Voltage endurance

Moreover, the demand for improved energy storage solutions has driven the development of power conditioning and management devices that endure higher operating voltages with greater reliability. The maximum energy an electrostatic capacitor can store is dependent on the dielectric permittivity and breakdown voltage of the insulating material separating the electrodes.^46–50^ For instance, commonly used polymer dielectric BOPP (biaxially oriented polypropylene) has relatively high electric breakdown (EBD) strength of ∼600 V μm^−1^.^51–53^ For electronic grade amorphous polymers, high breakdown strength has been previously associated with polar polymers.

**Fig. 3:**
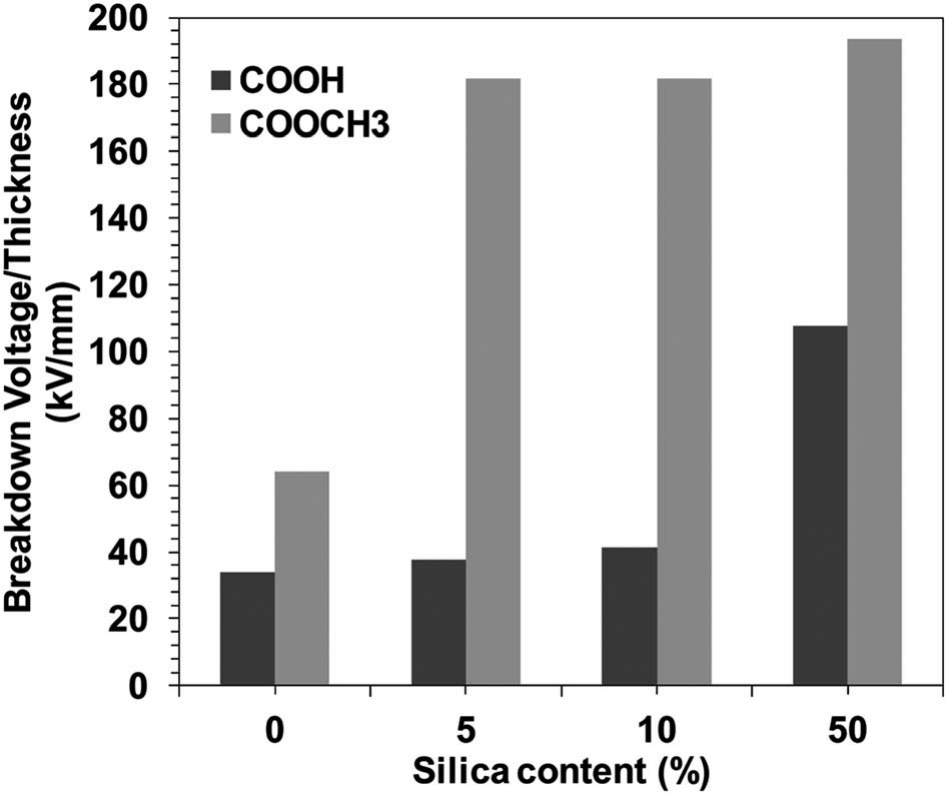
Breakdown voltage of biopolyimide with various silica contents.

The DC breakdown voltages of the BPI films with/without silica were measured. The BPI film thickness with and without silica were kept almost uniform, 27 ± 5 μm (Fig. S8†). Fig. 3 shows the silica concentration dependence of the DC breakdown voltage for BPI hybrids. It was observed that the introduction of the silica in the BPI matrix increases the DC breakdown voltage. However, the increase in the DC breakdown voltage was gradual in the case of COOH type BPI, *i.e.* up to 108 kV mm^−1^ (neat BPI COOH 34 kV mm^−1^). On the contrary, in the case of COOCH_3_ type BPI, the DC breakdown voltage for the neat sample was 64 kV mm^−1^ and increased up to more than 180 kV mm^−1^ at only 5 wt% silica concentration. This strongly indicates that the dispersion ability of silica within BPI matrix is different for COOH and COOCH_3_ type BPI. This behavior can be attributed to the depression in the percolation of electrical tree branching arose due to the uniform dispersion of the silica in the case of COOCH_3_ type BPI hybrids.

### Biopolyimide structure and silica hybrids performances

**Scheme 2:**
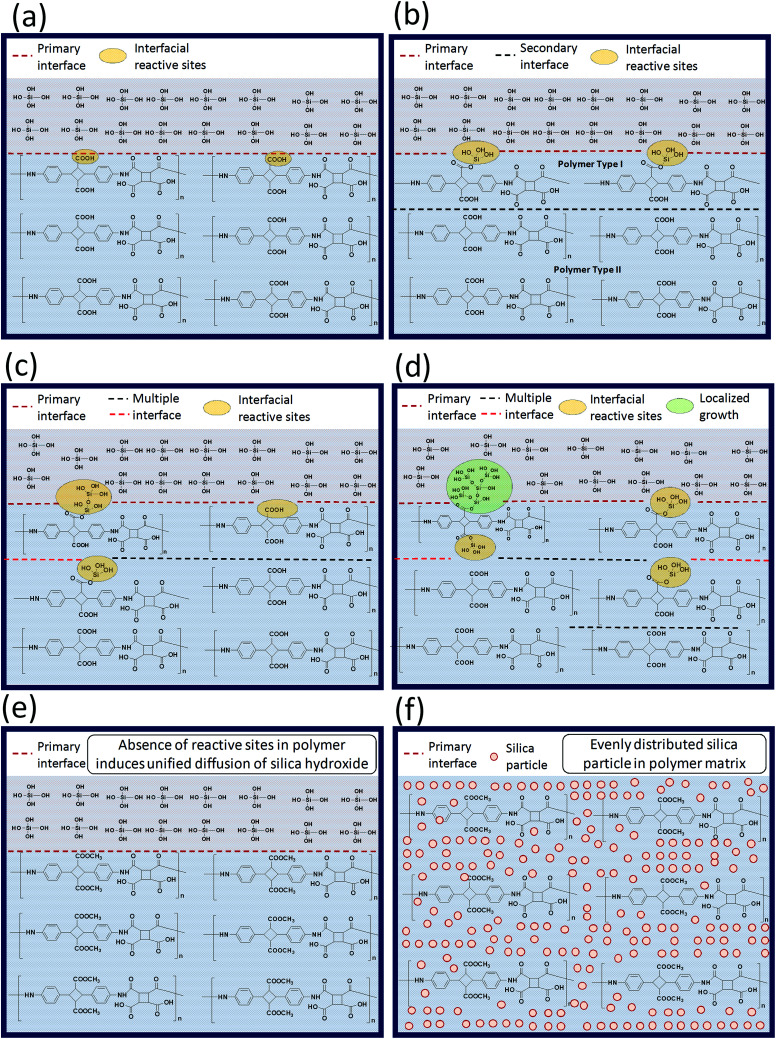
Schematic illustration of silica particle growth during polymerization with different biopolyimide micro-structures.

Variation in the molecular structure may affect the resulting BPI (neat) silica hybrids properties, especially when one of the BPI contains a functionalization reactive towards the TEOS sol–gel condensation. The schematic illustration proposed for expressing the difference in the reaction environment for the sol–gel condensation is as represented in the Scheme 2. When, COOH type poly(amic acid) mixed with TEOS for the sol–gel condensation (Scheme 2(a)), two important aspects arises (1) interface between TEOS solution and the poly(amic acid) solution) and, (2) interfacial reactive sites 4ATA–COOH group (4,4′-diamino-α-truxillic acid moiety) reacts with the TEOS during sol–gel reaction. These two aspects greatly control the sol–gel reaction progress. As reaction proceeds (Scheme 2(b)), the hydrolysis of TEOS produces Si(OH)_4_ which possess a reactivity towards the reaction with the 4ATA–COOH groups along the interface and get linked with the main polymer chain. Moreover, the reaction of interfacial 4ATA–COOH groups and/or localized growth of the silanols lead to the formation of multiple interfaces in the reaction medium, which retards the diffusion of the TEOS (Si(OH)_4_) in the reaction medium. Vigorous stirring of the reaction may lead to the forceful intermixing of the various interfaces and lead to the inter and/or intra-molecular linkage between the silanol molecules and the COOH type poly(amic acid) as shown in the (Scheme 2(c)). It is important to note that the interface shared between silanol molecules and over grown silanol-reacted COOH type poly(amic acid) grows much faster and lead to the non-uniform silica particle distribution along the polymer matrix (Scheme 2(d)) and reflected in the biopolyimide upon imidization. The non-uniform dispersion of the silica particle(s) in the polymer matrix lead to poor thermo-mechanical properties as well as opto-electrical properties. On the contrary, in the case of COOCH_3_ type poly(amic acid) the TEOS (or Si(OH)_4_) does not form multiple interfaces due to the absence of any reactive sites in the poly(amic acid) matrix. The absence of the reactive sites leads to a relatively more uniform diffusion of the silanol particles in the polymer matrix. The better dispersion of the silica particle in the polymer matrix leads to the superior thermo-mechanical, optical and electrical properties.

## Conclusions

A new kind of biopolyimide has been synthesized and thereby a systematic study has been performed to understand the effect of polymer microstructure on their silica hybrids performances. The hybrid films were obtained by the hydrolysis-polycondensation of TEOS, by optionally facilitating reaction with the biopolyimide backbone functionalization. It is important to note that the introduction of silica in the poly(amic acid) backbone affects the properties of biopolyimide films. COOCH_3_ type BPI enables uniform dispersion of silica particle in the BPI matrix, which leads to improved thermo-mechanical, optical as well as ultra-high voltage endurance capabilities. On the other hand, the COOH containing BPI enabled the reaction of silanols with the carboxylic group. It has been proposed that the localized silanol reaction proceeds with non-uniform silica particle dispersion in the polymer matrix and therefore, possess properties lower than the matrix with uniform particle dispersion. Overall, the technique of controlling the chemical bond between inorganic/organic materials is an important and useful means to modulate the properties of hybrid materials. Summarizing, these finely tuned hybrid films are one of the most promising candidates for flexible transparent substrate especially for high temperature (>350 °C) electronic and optoelectronic manufacturing processes.

## Conflicts of interest

The authors declare no conflicts of interests.

## Supplementary Material

RA-008-C8RA01965G-s001
