# Supplementary material for: Effects of biopolyimide molecular design on their silica hybrids thermo-mechanical, optical and electrical properties
Source: RSC Adv. 2018 Apr 16;8(25):14009–16. doi: 10.1039/c8ra01965g (PMC9079868; doi:10.1039/c8ra01965g)
Supplement: RA-008-C8RA01965G-s001 [file RA-008-C8RA01965G-s001.pdf]

Electronic Supplementary Material (ESI) for RSC Advances.
This journal is © The Royal Society of Chemistry 2018

Article for Polymer Chemistry

Supporting Information

Effect of biopolyimide molecular design on their silica hybrids thermo-mechanical, optical and electrical properties

S. Dwivedi,[a] S. Sakamoto,[a] S. Kato,[c] T. Mitsumata[b,c] and T. Kaneko[a,b]

---

[a]Graduate School of Advanced Science and Technology, Energy and Environment Area, Japan Advanced Institute of Science and Technology, 1-1 Asahidai, Nomi, Ishikawa 923-1292, Japan
[b]Japan Science and Technology, ALCA, Tokyo 102-0076, Japan
[c]Department of Materials Science & Technology, Faculty of Engineering, Niigata University, Ikarashi, Nishi-ku, Niigata 950-2181 Japan

† Corresponding Author
Tatsuo Kaneko
Email: kaneko@jaist.ac.jp
Electronic Supplementary Information (ESI) available: [Polyimide Synthesis and Characterization]. See DOI: 10.1039/x0xx00000x

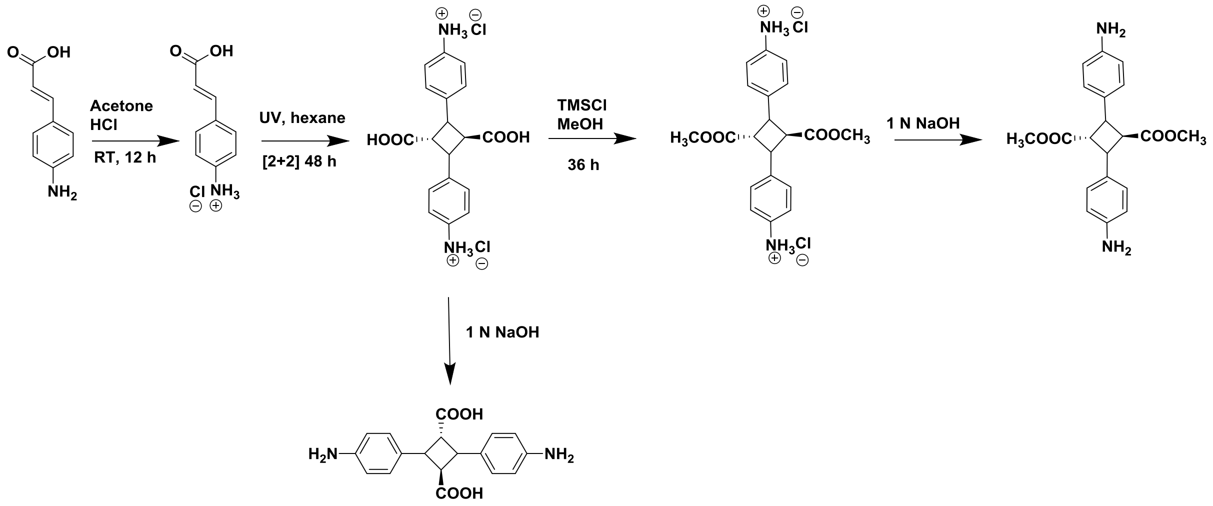

Scheme S1. Monomer synthesis from 4-aminocinnamic acid (4ACA)

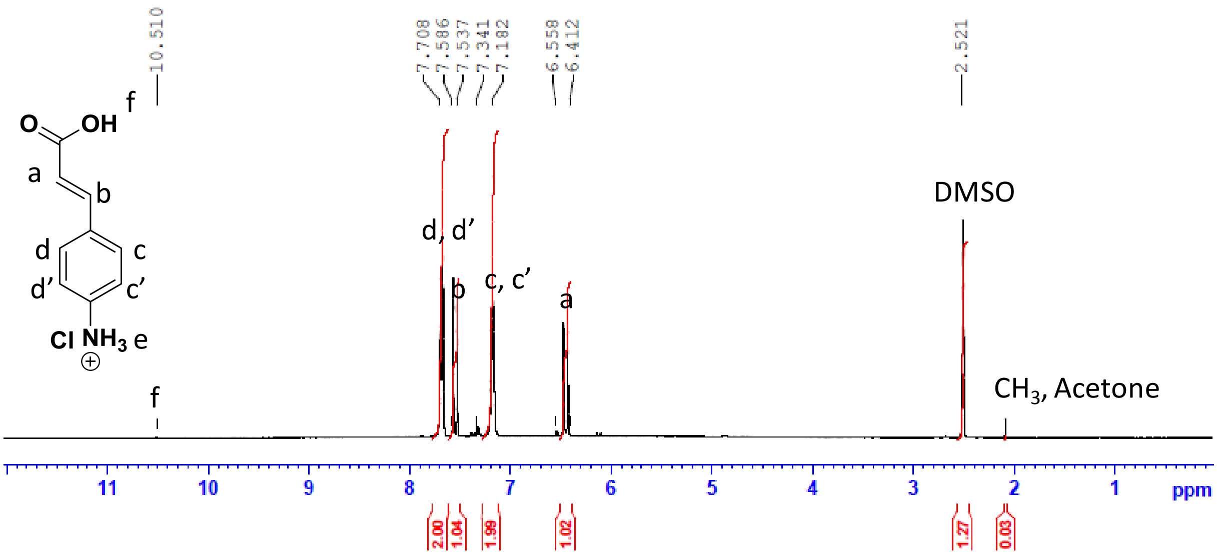

Figure S1. ^{1}H NMR characterization for 4ACA salt

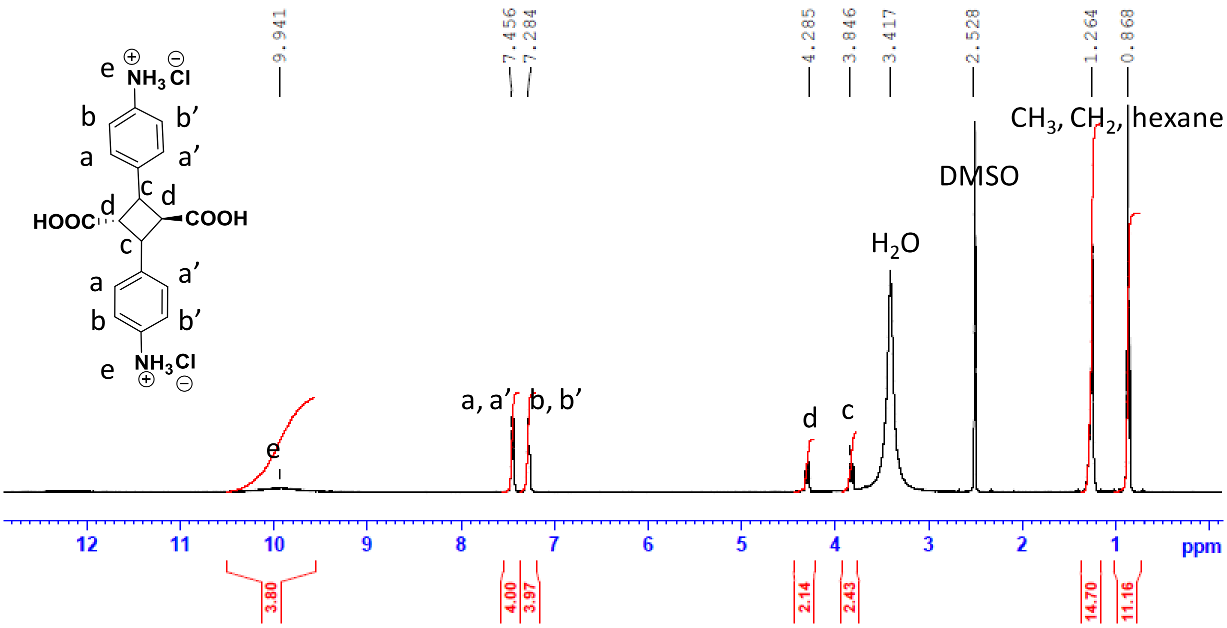

Figure S2. ^{1}H NMR characterization for 4ATA salt

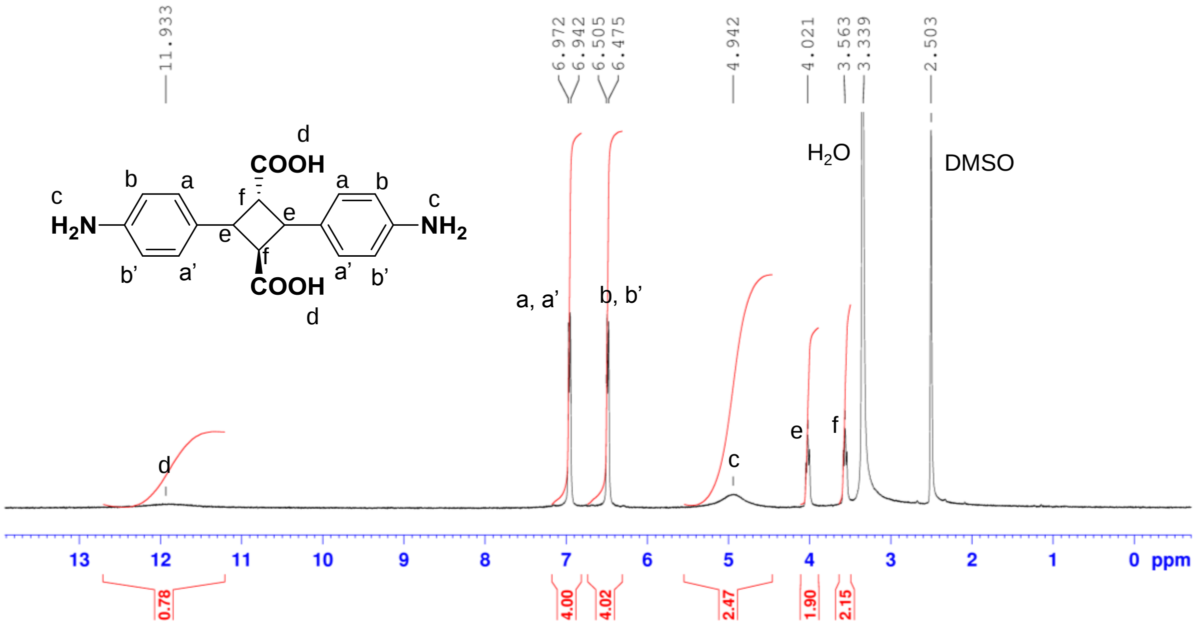

Figure S3. ^{1}H NMR characterization for 4ATA acid

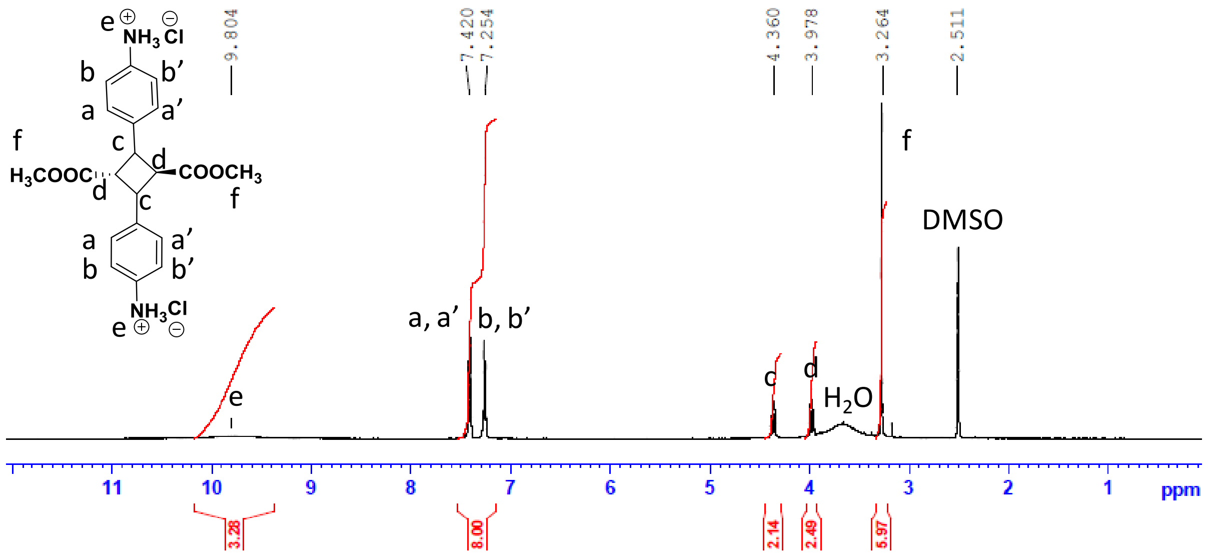

Figure S4. ^{1}H NMR characterization for 4ATA methyl ester salt

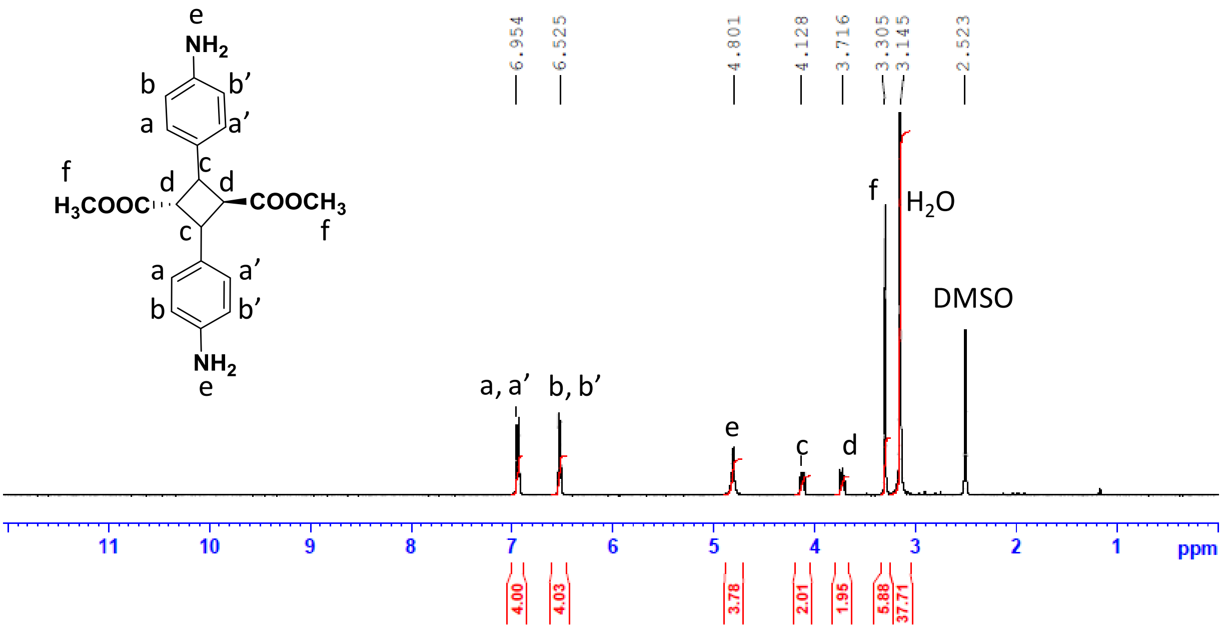

Figure S5. ^{1}H NMR characterization for 4ATA methyl ester

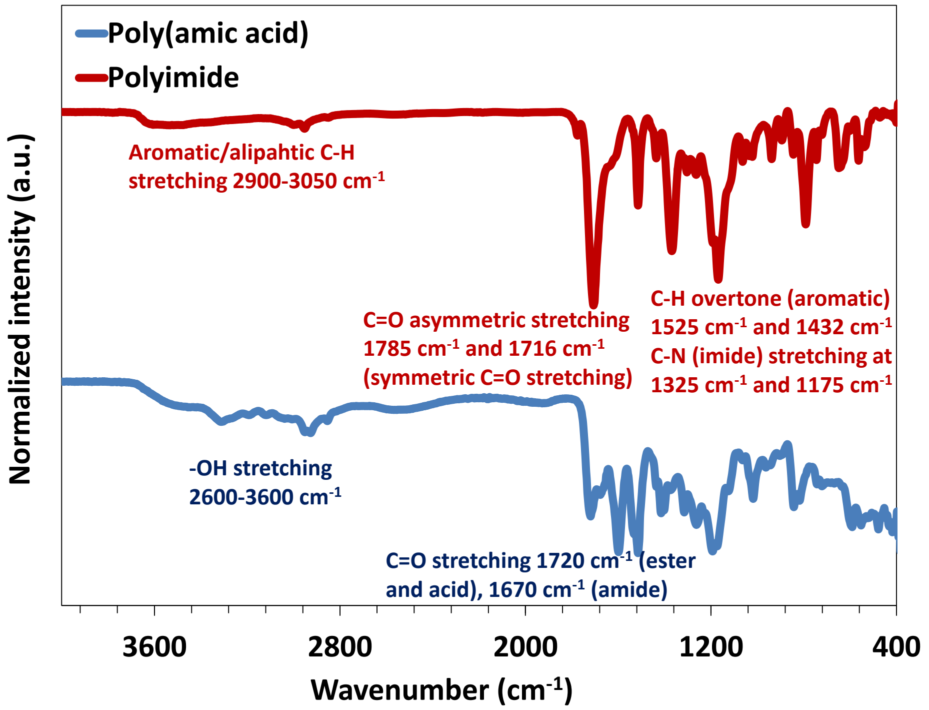

Figure S6. FTIR characterization for the prepared poly(amic acid) and polyimide

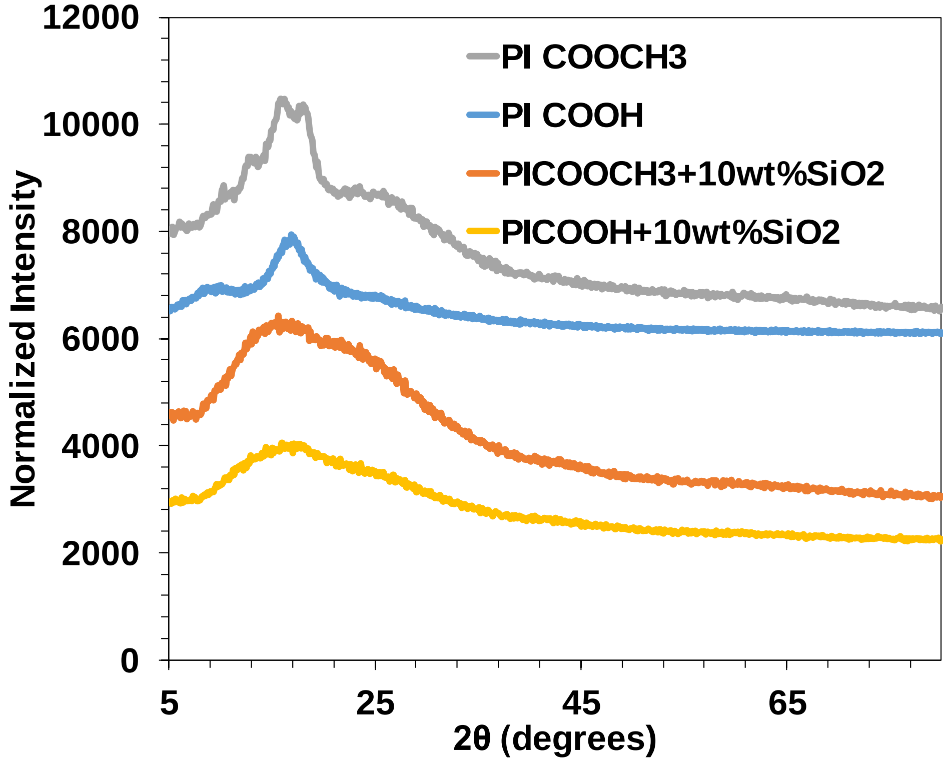

Figure S7. XRD patterns for the neat biopolyimide and after introducing 10 wt% of silica

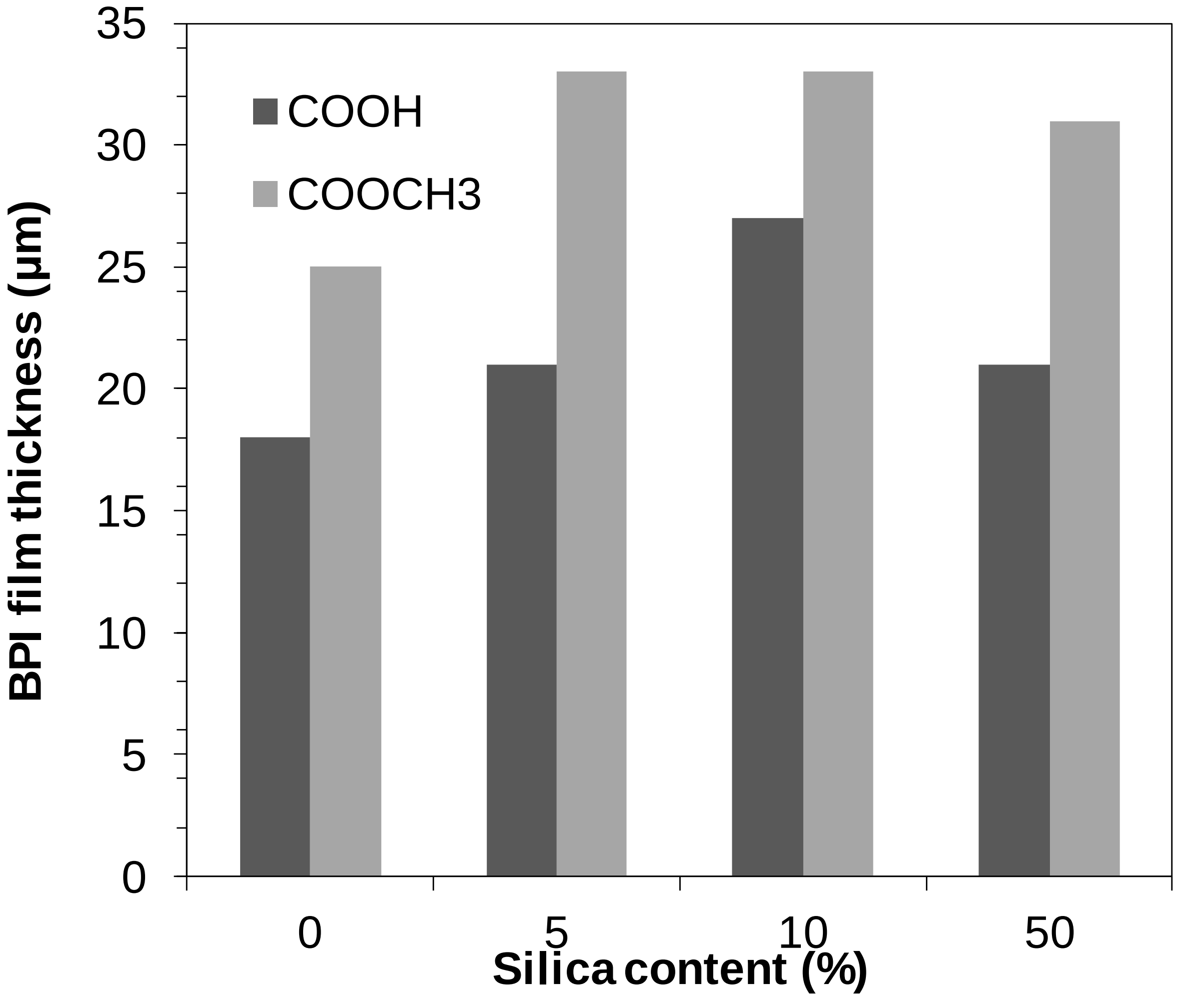

Figure S8. BPI film thickness with/without silica used for the electric breakdown voltage test
